# Reviewers in 2022

**DOI:** 10.1098/rsbl.2023.0095

**Published:** 2023-03-15

**Authors:** David Beerling

**Affiliations:** Editor-in-Chief

As has become a welcome annual tradition *Biology Letters* is pleased to take this opportunity to thank all our peer reviewers who worked with us in 2022. This includes many co-reviewers, who are often early-career researchers and students getting valuable experience in the science publishing process as well as providing significant help to the journal. On behalf of the entire Editorial Board, I would like to say how much we appreciate your insights, critiques and suggestions that go into each peer reviewer report. Now many of our readers can see your efforts too since launching our optional Transparent Peer Review process last year—we hope these also act as tools for future referees.

We know that most reviewers value recognition of their efforts by the journal and through services, such as Publons, which is integrated with *Biology Letters*. To provide an opportunity for recognition, we also list the names of all reviewers (unless they have opted out). This article is made permanent and citable by its digital object identifier (DOI). We encourage you to quote your contribution in your applications for tenure and grants or other forms of research assessment. Where you have provided it, we have included your ORCID, so your contribution can be unambiguously assigned to you.

The *Biology Letters* team looks forward to working with you all again.

Abram P
Adamo S https://orcid.org/0000-0002-5973-571X
Adams B https://orcid.org/0000-0002-5857-175X
Adema C https://orcid.org/0000-0002-6250-5499
Aditya G
Adkins-Regan E
Agrawal A https://orcid.org/0000-0003-0095-1220
Aharon-Rotman Y
Alaasam V https://orcid.org/0000-0003-1670-6780
Albon S
Alibardi L
Alizon S https://orcid.org/0000-0002-0779-9543
Alizon S https://orcid.org/0000-0002-0779-9543
Allen C
Almeida-Santos S
Amar A
Amaya-Márquez M https://orcid.org/0000-0001-6135-8063
Amendt J
Ancillotto L https://orcid.org/0000-0002-8774-0671
Andrew S
Antoniazzi M
Arai T https://orcid.org/0000-0001-9440-7933
Archer C https://orcid.org/0000-0003-2501-425X
ARIA C https://orcid.org/0000-0002-0567-1730
Assis V https://orcid.org/0000-0003-0326-4397
Atutey E
Auge H
Avarguès-Weber A https://orcid.org/0000-0003-3160-6710
Aviles J https://orcid.org/0000-0002-1463-8393
Avilés L https://orcid.org/0000-0002-9609-2952
Baier L https://orcid.org/0000-0002-0327-0378
Bailey J https://orcid.org/0000-0002-6365-8120
Balbuena J
Balfour N https://orcid.org/0000-0002-2426-3898
Ballen G
Barbeito C
Barker AJ
Barnes E
Barraquand F https://orcid.org/0000-0002-4759-0269
Barreto R
Bastos A
Beck K
Becker D https://orcid.org/0000-0003-4315-8628
Beedholm K
Beermann J
Behrens J https://orcid.org/0000-0002-0136-9681
Behringer D https://orcid.org/0000-0001-5244-471X
Bell J https://orcid.org/0000-0001-9996-945X
Bentley M
Bentz E
Berhman E
Bernotiene R
Bertram M https://orcid.org/0000-0001-5320-8444
Bierbach D
Bitzer R
Bjelica V https://orcid.org/0000-0002-3625-4470
Bladon A
Blakey R https://orcid.org/0000-0002-6654-5703
Bleicher S https://orcid.org/0000-0001-8727-5901
Bode A
Boesch C
Bolt L https://orcid.org/0000-0002-8275-6543
Borg B
Bose A https://orcid.org/0000-0001-5716-0097
Bourke A
Boyles J https://orcid.org/0000-0003-1494-4515
Brakefield P
Brand CM
Braude S
Bravo I https://orcid.org/0000-0003-3389-3389
Breed G
Breuner C
Brigham R
Brink K https://orcid.org/0000-0001-6630-1525
Briscoe A https://orcid.org/0000-0001-8514-4983
Bronstein J
Brown G
Brown K https://orcid.org/0000-0001-5235-5928
Brunton B https://orcid.org/0000-0002-4831-3466
Brunton D
Bulmer M https://orcid.org/0000-0003-1970-9485
Burkett-Cadena N https://orcid.org/0000-0001-6168-1637
Burness G
Busch J
Busia L https://orcid.org/0000-0001-9542-9130
Butovskaya M
Byrne M https://orcid.org/0000-0002-8902-9808
C. Lopes P https://orcid.org/0000-0001-5170-3230
Caliman A
Camp J
Campiao K
Canário A https://orcid.org/0000-0002-6244-6468
Cano FJ https://orcid.org/0000-0001-5720-5865
Canuti M
Capobianco A https://orcid.org/0000-0002-6096-3875
Cappa F https://orcid.org/0000-0002-3510-8885
Cardoso G https://orcid.org/0000-0001-6258-1881
Carlson J
Carlucci R
Carpenter K
Carrier T https://orcid.org/0000-0001-7885-184X
Carter G https://orcid.org/0000-0001-6933-5501
Carve M https://orcid.org/0000-0003-2649-8727
Carver S https://orcid.org/0000-0002-3579-7588
Cattadori I https://orcid.org/0000-0001-6618-316X
Cervo R https://orcid.org/0000-0002-0405-6402
Chakraborty S https://orcid.org/0000-0003-3801-1605
Chan B https://orcid.org/0000-0001-9479-024X
Chang C-H
Chapman J https://orcid.org/0000-0002-7475-4441
Charabidze D https://orcid.org/0000-0002-9935-1956
Charlesworth D https://orcid.org/0000-0002-3939-9122
Chen G
Cheney K https://orcid.org/0000-0001-5622-9494
Chirichella R
Cilia G
Cimino M
Civetta A
Civitello D https://orcid.org/0000-0001-8394-6288
Clarindo W
Clark C https://orcid.org/0000-0002-6311-9768
Clegg S
Cohen J
Cole B https://orcid.org/0000-0002-9350-8457
Collins C
Colon F
Condamine F https://orcid.org/0000-0003-1673-9910
Connor-Stroud F https://orcid.org/0000-0003-3981-8654
Conover J https://orcid.org/0000-0002-3558-6000
Convey P https://orcid.org/0000-0001-8497-9903
Conway K https://orcid.org/0000-0002-0161-8385
Cook ASCP
Cook C https://orcid.org/0000-0003-3310-2161
Coombes M
Cornulier T
Cossey S https://orcid.org/0000-0002-6255-3423
Costa F https://orcid.org/0000-0001-6218-9660
Cotter S https://orcid.org/0000-0002-3801-8316
Coughlin D https://orcid.org/0000-0001-9816-2742
Counterman B https://orcid.org/0000-0003-2724-071X
Cozzi G
Cram D https://orcid.org/0000-0002-8790-8294
Crespi B https://orcid.org/0000-0002-0362-392X
Cribellier A https://orcid.org/0000-0001-5113-7531
Croft D https://orcid.org/0000-0001-6869-5097
Cronk L
Cruz-Neto A
Cuesta JA
Culina A https://orcid.org/0000-0003-2910-8085
Cullen T https://orcid.org/0000-0002-4261-1323
Currano E
Czaczkes T https://orcid.org/0000-0002-1350-4975
Dallinger R
Dalziell A
Dammann P https://orcid.org/0000-0002-0624-3965
Dani F https://orcid.org/0000-0002-2939-2103
Darragh K
Daura-Jorge FG
de Margerie E https://orcid.org/0000-0002-5380-3355
de Oliveira Ramalho M
de Vos J
Deangelis D
Dechow P https://orcid.org/0000-0002-0204-4774
Degen J
Degrange F
Delhey K https://orcid.org/0000-0001-5190-5406
DeLong J https://orcid.org/0000-0003-0558-8213
Delorme N
Derégnaucourt S
Derocher A
DeRuiter S
Diamond K
Diaz F
Dick E
Dickerson B
Ding Q
Dionisio F https://orcid.org/0000-0002-3653-1511
Dobata S
Doherty J-F https://orcid.org/0000-0003-4766-9417
Doll J
Dowdy N https://orcid.org/0000-0002-5453-2569
Dreyer N https://orcid.org/0000-0002-1391-1642
Dubiec A
Duffy M https://orcid.org/0000-0002-8142-0802
Dugas MB
Dunlap A https://orcid.org/0000-0003-2429-5758
Dunn R
Durbach I
Dyer A https://orcid.org/0000-0002-2632-9061
Dzialowski E
Earl J
Edinborough K
Edwards P https://orcid.org/0000-0003-3658-5146
Elemans C
Elmore S
Elvidge C
Endler J https://orcid.org/0000-0002-7557-7627
Endo A https://orcid.org/0000-0001-6377-7296
Engelberth J
Ernst J
Estrada-Peña A
Evans A
Evans J https://orcid.org/0000-0002-2603-6832
Fabbri M https://orcid.org/0000-0002-1257-1594
Fang S
Farine D https://orcid.org/0000-0003-2208-7613
Farnkopf I https://orcid.org/0000-0002-7800-2335
Feder J https://orcid.org/0000-0002-3087-7298
Fenton B
Fiedler K
Fields P https://orcid.org/0000-0003-2959-2524
Finkbeiner S https://orcid.org/0000-0001-8525-6141
Fisher D
Fleagle J
Forstmeier W https://orcid.org/0000-0002-5984-8925
Foster K
Foster W
Foulkes N
Franchi GA
Franco-Trecu V
Frankish C
Freckelton M https://orcid.org/0000-0001-6451-6020
Freeman M https://orcid.org/0000-0002-7801-220X
Friesen C https://orcid.org/0000-0001-5338-7454
Frost F
Fuess L https://orcid.org/0000-0003-0197-7326
Gabor C https://orcid.org/0000-0001-7584-1451
Gaillard J-M
Gallagher A
Garfield Z https://orcid.org/0000-0002-1547-1492
Garland E https://orcid.org/0000-0002-8240-1267
Garm A http://orcid.org/0000-0002-2080-735X
Garner T https://orcid.org/0000-0002-0336-9706
Genner M https://orcid.org/0000-0003-1117-9168
Gerken A
Ghiselli F https://orcid.org/0000-0002-1680-8616
Gillies N https://orcid.org/0000-0002-9950-609X
Giray T
Giroud S https://orcid.org/0000-0001-6621-7462
Glendinning J
Glennon E https://orcid.org/0000-0001-9540-1998
Goiran C https://orcid.org/0000-0002-1649-4418
Golubović A
Gonzalez Betancourt VH https://orcid.org/0000-0002-4146-1634
Goodman S
Goodman S
Gotcha N https://orcid.org/0000-0003-1292-4991
Goto S https://orcid.org/0000-0002-4431-7531
Goumas M https://orcid.org/0000-0001-9338-2412
Grande L
Gray D https://orcid.org/0000-0002-4439-561X
Gray J https://orcid.org/0000-0001-9079-7962
Green A
Greppi C https://orcid.org/0000-0001-5827-3448
Gridi-Papp M http://orcid.org/0000-0003-4630-7510
Griffith O https://orcid.org/0000-0001-9703-7800
Gruber T https://orcid.org/0000-0002-6766-3947
Grunst M https://orcid.org/0000-0002-3425-4020
Guerra PA
Guleria P https://orcid.org/0000-0001-5235-4829
Gunderson A
Gustison M
Gutierrez J-C
Gutiérrez-Cánovas C
Haan NL
Hall J https://orcid.org/0000-0002-5587-3402
Hall M https://orcid.org/0000-0002-4738-203X
Hambäck P
Han K-A
Hao Y
Hara Y https://orcid.org/0000-0001-6647-6595
Harmon JP
Harris R https://orcid.org/0000-0002-4412-2275
Harvey E
Hauber M https://orcid.org/0000-0003-2014-4928
Hawley D
Hechavarria J https://orcid.org/0000-0001-9277-2339
Hedgespeth M https://orcid.org/0000-0002-3683-3114
Hedlund J
Helantera H https://orcid.org/0000-0002-6468-5956
Hellgren O
Heltai M
Hempel de Ibarra N https://orcid.org/0000-0002-0859-8217
Heneberg P http://orcid.org/0000-0002-0703-951X
Henrique G https://orcid.org/0000-0002-6039-0771
Henry L https://orcid.org/0000-0002-3130-4643
Henrys P http://orcid.org/0000-0003-4758-1482
Henshaw J https://orcid.org/0000-0001-7306-170X
Henzi P https://orcid.org/0000-0001-6175-1674
Herbert N
Hernandes FA
Hernández JC https://orcid.org/0000-0002-1539-1783
Heyman T https://orcid.org/0000-0003-0565-441X
Hill G
Hiltunen T https://orcid.org/0000-0001-7206-2399
Hines H https://orcid.org/0000-0003-0299-5569
Ho L
Ho M https://orcid.org/0000-0002-1454-4768
Hodges-Simeon C
Hofmann H https://orcid.org/0000-0002-3335-330X
Holekamp K https://orcid.org/0000-0001-5471-1076
Hoogenboom M
Hopper L https://orcid.org/0000-0001-5953-3264
Horner J
Hovestadt T https://orcid.org/0000-0001-7368-6013
Huber N https://orcid.org/0000-0002-2371-5645
Humble E
Hunter K
Huss J
Hutchins M
Ibarbalz F https://orcid.org/0000-0003-4326-5490
Ilinsky Y
Illing B
Inouye D
Iriarte-Diaz J https://orcid.org/0000-0003-3566-247X
Irisarri I https://orcid.org/0000-0002-3628-1137
Irmis R
Isaac N
Jarčuška B https://orcid.org/0000-0002-0654-9171
Jiang P
Jiao H
Johnson K
Johnson M
Johnson M
Joyce P https://orcid.org/0000-0003-1058-7901
Käfer H
Kageyama D https://orcid.org/0000-0002-9026-9825
Kalinkat G https://orcid.org/0000-0003-3529-5681
Kaltz O
Kamath A https://orcid.org/0000-0002-4012-0483
Kanna R
Katsis A
Kavaliers M https://orcid.org/0000-0003-2292-5963
Keiser C https://orcid.org/0000-0002-4936-7810
Kelly D
Kelly J
Kempenaers B https://orcid.org/0000-0002-7505-5458
Kendal J https://orcid.org/0000-0001-8644-3117
Ketterson E
Khadempour L https://orcid.org/0000-0002-7043-3720
Kiełkiewicz M
Kiffner C
Kim A
Kingsolver J https://orcid.org/0000-0002-9839-0208
Kinziger A
Kitahara M
Klug C https://orcid.org/0000-0002-4099-7453
Kobayashi K
Koch J https://orcid.org/0000-0002-6654-7370
Komar N
Kong L
Koprivnikar J https://orcid.org/0000-0001-8410-1041
Kot I
Kotlík P https://orcid.org/0000-0001-9429-0667
Krajnak K
Kralj-Fiser S
Kramer J https://orcid.org/0000-0003-2155-9295
Kranstauber B
Kretschmer R
Kriengwatana B https://orcid.org/0000-0002-4258-883X
Krishnan N
Kristjánsson B
Kronauer D https://orcid.org/0000-0002-4103-7729
Kuntner M
Kusch J https://orcid.org/0000-0003-0078-5621
La Fortezza M https://orcid.org/0000-0002-4485-9840
Lacasa L
Lacour M
Lagos N
Lailvaux S https://orcid.org/0000-0002-2737-8682
Lamacque L https://orcid.org/0000-0001-9414-0941
Lane S https://orcid.org/0000-0002-3797-3178
Lankheet M https://orcid.org/0000-0002-8261-2187
LaSharr T
Latombe G
Laubach ZM https://orcid.org/0000-0003-2614-139X
Laurin M
Lees A
Lehmann K https://orcid.org/0000-0003-2690-7332
Lemmers S
Lendvai Á https://orcid.org/0000-0002-8953-920X
Leponce M
Lessios H
Levesque D https://orcid.org/0000-0003-0132-8094
Levis N https://orcid.org/0000-0001-7650-2371
Lewis C https://orcid.org/0000-0002-3564-2906
Li Q
Liang W https://orcid.org/0000-0002-0004-9707
Libbrecht R https://orcid.org/0000-0003-4397-000X
Lihoreau M https://orcid.org/0000-0002-2463-2040
Liu C
Livesley SJ
Llaurens V
LoPresti E
Lovas-Kiss A
Low F https://orcid.org/0000-0001-7136-9997
Lu A
Luizzi V
Lynch H https://orcid.org/0000-0002-9026-1612
MacCormack T
MacDougall-Shackleton S https://orcid.org/0000-0001-8518-765X
Macgregor C https://orcid.org/0000-0001-8281-8284
Machado F
Machlus K
MacLean H https://orcid.org/0000-0003-0982-3209
Maclean I https://orcid.org/0000-0001-8030-9136
Maitra S
Mallet J
Malone J
Marcantonio M https://orcid.org/0000-0003-3896-2355
Marcus J
Marshall K https://orcid.org/0000-0002-6991-4957
Marsico T
Marsland S https://orcid.org/0000-0002-9568-848X
Martin MV
Martin K
Martínez-Freiría F
Martin-Ordas G https://orcid.org/0000-0002-5221-9181
Mason R https://orcid.org/0000-0002-2725-2449
Massa B
Matsuzawa T https://orcid.org/0000-0002-8147-2725
Matthiopoulos J https://orcid.org/0000-0003-3639-8172
Maturana C https://orcid.org/0000-0002-4427-8093
Matyas G
Mayhew J
Mazzoni V https://orcid.org/0000-0003-4036-5322
McClelland G
McCracken A
McCracken K
McGraw K https://orcid.org/0000-0001-5196-6620
McHuron E https://orcid.org/0000-0003-3147-2628
Mejias C https://orcid.org/0000-0002-2950-3651
Mendiola S
Mendl M https://orcid.org/0000-0002-5302-1871
Merilä J https://orcid.org/0000-0001-9614-0072
Metcalfe N https://orcid.org/0000-0002-1970-9349
Miličić M https://orcid.org/0000-0002-3154-660X
Miller R
Mitchell A
Mithöfer A https://orcid.org/0000-0001-5229-6913
Moerman F https://orcid.org/0000-0002-5164-0978
Mongeau J-M https://orcid.org/0000-0002-3292-6911
Monteith K
Moore P https://orcid.org/0000-0001-9802-7217
Morfin N
Morley S https://orcid.org/0000-0002-7761-660X
Morris C
Morrison C https://orcid.org/0000-0002-4293-2717
Morrissey C
Moscovitz S
Mounce R
Munday P
Mustonen A-M
Mutshinda CM https://orcid.org/0000-0001-9671-7812
Myers A
Myosho T
Nakata T https://orcid.org/0000-0002-0378-2242
Navara K https://orcid.org/0000-0002-1250-8480
Navarro J https://orcid.org/0000-0002-5756-9543
Nazarova G
Nelsen D
Nelson R
Neumann P https://orcid.org/0000-0001-5163-5215
Nicolaï M https://orcid.org/0000-0002-9570-0311
Niewiarowski P
Nilsson A https://orcid.org/0000-0002-3541-9835
Nityananda V https://orcid.org/0000-0002-2878-2425
Nokelainen O
Nonacs P https://orcid.org/0000-0003-3010-0777
Nord A https://orcid.org/0000-0001-6170-689X
Norin T https://orcid.org/0000-0003-4323-7254
Norris, D https://orcid.org/0000-0003-4874-1425
Novak C
Nunn C https://orcid.org/0000-0001-9330-2873
Nyamukondiwa C https://orcid.org/0000-0002-0395-4980
Ochola L
O'Hara R https://orcid.org/0000-0001-9737-3724
Okada Y
Ollerton J
Onyango E https://orcid.org/0000-0003-0116-4923
Oosthuizen C
Oppel S
Ord T https://orcid.org/0000-0002-2608-2150
Ornelas-García CP
Ortega-Hernández J https://orcid.org/0000-0002-6801-7373
Osakabe M
Osvath M https://orcid.org/0000-0002-7873-0930
Otaki J
Othman S
Owens A https://orcid.org/0000-0001-7212-2275
Oyserman D https://orcid.org/0000-0002-2727-2167
Ożarowska A
Pace R https://orcid.org/0000-0002-3126-635X
Palazzesi L
Palfalvi G https://orcid.org/0000-0002-4526-8954
Pálsson S
Pandey A
Papafitsoros K https://orcid.org/0000-0001-9691-4576
Papaj D
Parker J
Parker M
Pascall D https://orcid.org/0000-0002-7543-0860
Patrick S
Pearse I https://orcid.org/0000-0001-7098-0495
Pelster B
Peña-Kairath C
Peona V https://orcid.org/0000-0001-5119-1837
Pepi A
Pérez L
Peris D https://orcid.org/0000-0003-4074-7400
Pespeni M https://orcid.org/0000-0001-5447-6678
Petanidou T
Petrullo L https://orcid.org/0000-0001-6464-3177
Pilastro A https://orcid.org/0000-0001-9803-6308
Pilowsky P
Pincebourde S
Platt B
Plazzi F
Plum K
Poggiale J-C https://orcid.org/0000-0002-3888-0096
Porras M
Potter D
Poulin R https://orcid.org/0000-0003-1390-1206
Prins H
Procenko O
Pulgar J
Putman B
Pyron M
Qadri M
Quigley C https://orcid.org/0000-0002-7522-4426
Quijón P
Quinn J
Raia P https://orcid.org/0000-0002-4593-8006
Rands S https://orcid.org/0000-0002-7400-005X
Ranke P https://orcid.org/0000-0003-3757-8626
Rasmussen JJ
Razanajatovo M https://orcid.org/0000-0001-9181-7363
Rebelo J https://orcid.org/0000-0002-8662-7378
Reed T
Reimer J
Reisinger R https://orcid.org/0000-0002-8933-6875
Reyne B https://orcid.org/0000-0002-4899-1240
Reznick D https://orcid.org/0000-0002-1144-0568
Richardson K
Richardson M
Ritchie M
Rochman C
Rodrigues Simões T
Rodriguez A https://orcid.org/0000-0001-7882-135X
Rodriguez-Cabal M
Rodriguez-Lanetty M https://orcid.org/0000-0003-3007-2841
Rogers L https://orcid.org/0000-0002-9956-1769
Rosén A
Rosenheim J
Roth E
Rouse A https://orcid.org/0000-0003-3504-5906
Roux C
Rowe C
Rowe T
Rozaimi M https://orcid.org/0000-0001-6631-8677
Rozen D https://orcid.org/0000-0002-7772-0239
Rubino F
Rueger T https://orcid.org/0000-0002-2105-6412
Ruf T
Rushing C https://orcid.org/0000-0002-9283-6563
Ryan M https://orcid.org/0000-0002-6381-9545
Sabandal P
Saccheri I https://orcid.org/0000-0003-0476-2347
Sagi A
Sagona S
Sakakura Y
Sakauchi K
Sandercock B
Sane M
Sankey DWE https://orcid.org/0000-0002-6363-8023
Santidrián P
Santin J https://orcid.org/0000-0003-1308-623X
Satoh N https://orcid.org/0000-0002-4480-3572
Schaffeld T
Scharf I https://orcid.org/0000-0002-8506-7161
Schausberger P https://orcid.org/0000-0002-1529-3198
Scheyer T https://orcid.org/0000-0002-6301-8983
Schino, G.https://orcid.org/0000-0002-5011-4896
Schipper A
Schlupp I https://orcid.org/0000-0002-2460-5667
Schmoll T https://orcid.org/0000-0003-3234-7335
Scholz B
Schuch AP
Schülke O https://orcid.org/0000-0003-0028-9425
Scrosati R
Sefc K https://orcid.org/0000-0001-8108-8339
Segura D
Sekar M
Semple J
Sepulveda M https://orcid.org/0000-0002-1403-176X
Sequeira A
Serrano E
Serrao JE https://orcid.org/0000-0002-0477-4252
Seymoure B
Shankar A https://orcid.org/0000-0002-3043-6126
Shelly T
Sherley R https://orcid.org/0000-0001-7367-9315
Shimadzu H https://orcid.org/0000-0003-0919-8829
Shimeta J https://orcid.org/0000-0002-7003-7832
Shine R https://orcid.org/0000-0001-7529-5657
Shirley M
Siddiqui R https://orcid.org/0000-0001-9646-6208
Siddiqui S
Sidor C
Silby M
Simons M
Singer M
Singer M
Sinu P https://orcid.org/0000-0001-7391-7133
Skevington J
Slade J
Smallegange I
Smith AR https://orcid.org/0000-0002-4291-6928
Smith D
Smith H
Snyder B
Sokolova I
Solis A https://orcid.org/0000-0003-1736-3767
Sommer-Trembo C https://orcid.org/0000-0001-9952-2906
Sørensen M
Soroka M
Sota T https://orcid.org/0000-0002-0400-9147
Sparkman A https://orcid.org/0000-0002-3504-6426
Srivathsan A
Steffen J https://orcid.org/0000-0002-0106-2452
Steidle J https://orcid.org/0000-0003-2386-3063
Steinauer M
Stewart J https://orcid.org/0000-0001-6851-2289
Stier A https://orcid.org/0000-0002-5445-5524
Strand A
Strelin M
Suarez A https://orcid.org/0000-0002-2257-3366
Suchting R
Sudyka J
Sugahara R
Sun J https://orcid.org/0000-0002-9465-3672
Sustaita D https://orcid.org/0000-0001-9932-909X
Swearer S
Swierk L https://orcid.org/0000-0001-7897-0275
Szydlo W
Takahashi T https://orcid.org/0000-0003-1770-2116
Talbott K https://orcid.org/0000-0003-1974-1899
Talwar C https://orcid.org/0000-0002-7455-4423
Tanner J https://orcid.org/0000-0002-7047-7256
Tattersall G
Teitelbaum C https://orcid.org/0000-0001-5646-3184
Thogmartin W https://orcid.org/0000-0002-2384-4279
Thompson A https://orcid.org/0000-0002-1384-2229
Thompson K https://orcid.org/0000-0002-1487-3254
Thorley J
Tilbrook A
Tougaard J https://orcid.org/0000-0002-4422-7800
Townsend A https://orcid.org/0000-0002-2452-1594
Triebskorn R
Trumbo S https://orcid.org/0000-0002-4455-4211
Tudorache C https://orcid.org/0000-0002-6208-9608
Turner P
V. Mohan A
Valente D https://orcid.org/0000-0001-6086-5135
Van Damme R https://orcid.org/0000-0002-4585-6940
van der Niet T
van der Wal J https://orcid.org/0000-0002-6441-3598
Van Doorslaer K
van Erp J https://orcid.org/0000-0001-6888-0057
Van Goor J https://orcid.org/0000-0001-7498-9315
van Leeuwen J
van Thiel J
van Velzen E
Van Wassenbergh S https://orcid.org/0000-0001-5746-4621
Vandeleest J https://orcid.org/0000-0001-7880-0700
Varkoulis A
Velotta J
Verbe A
Verhulst S https://orcid.org/0000-0002-1143-6868
Viegas I http://orcid.org/0000-0003-2589-2212
Vincze O
Vinther J https://orcid.org/0000-0002-3584-9616
Viranta S https://orcid.org/0000-0003-4105-530X
Vizueta J https://orcid.org/0000-0003-0139-3013
Vogels R
Vogler A
Völter C
Vorburger C https://orcid.org/0000-0002-3627-0841
Voulgaris K
Wagner D
Waldvogel A-M
Walker S
Walters J
Walton R https://orcid.org/0000-0002-2258-1374
Wang B
Wang R https://orcid.org/0000-0003-4652-2149
Wang S https://orcid.org/0000-0002-0496-1728
Wanner S
Ward M https://orcid.org/0000-0001-6651-4715
Warren R
Wascher C
Webster M
Wenne R
Westbury M https://orcid.org/0000-0003-0478-3930
Whalan S
Whalen CD
Whelan N
White S
Whitehead H https://orcid.org/0000-0001-5469-3429
Whiting M https://orcid.org/0000-0002-4662-0227
Whittington C https://orcid.org/0000-0001-5765-9699
Wiberg A https://orcid.org/0000-0002-8074-8670
Wiebe K
Wilkinson G https://orcid.org/0000-0001-7799-8444
Williams H https://orcid.org/0000-0003-0947-6243
Wilson C
Wilson K https://orcid.org/0000-0002-6356-1898
Wilsterman K https://orcid.org/0000-0001-7262-9754
Witte K
Woelfing B
Womack M https://orcid.org/0000-0002-3346-021X
Woodborne S
Xavier R https://orcid.org/0000-0001-8800-3924
Yannic G https://orcid.org/0000-0002-6477-2312
Ye J
Yorzinski J https://orcid.org/0000-0002-4193-6695
Yoshido A https://orcid.org/0000-0003-0343-2709
Young H https://orcid.org/0000-0003-0449-8582
Yu H https://orcid.org/0000-0003-4558-6212
Zagalska-Neubauer M https://orcid.org/0000-0002-9738-1393
Zajitschek F https://orcid.org/0000-0001-6010-6112
Zalucki M https://orcid.org/0000-0001-9603-7577
Zelditch M https://orcid.org/0000-0002-5006-6679
Zhang H https://orcid.org/0000-0002-2674-3035
Zhang Y
Zöttl M https://orcid.org/0000-0002-5582-2306
Zou Y
Zulfiqar S
Zuschin M
Zwolak R https://orcid.org/0000-0002-7665-5033

